# Submassive Pulmonary Thromboembolism in a Patient with Thrombocytopenia: Therapeutic Challenge

**DOI:** 10.1155/2019/1919401

**Published:** 2019-01-22

**Authors:** Ronni Andrea Muñoz Tovar, Luis Carlos Alvarez Perdomo, Sandra Milena Rojas Molina, Silvana Jimenez Salazar

**Affiliations:** ^1^Critical Care, Hospital Universitario Hernando Moncaleano Perdomo, Neiva, Huila, Colombia; ^2^Internal Medicine, Hospital Universitario Hernando Moncaleano Perdomo, Neiva, Huila, Colombia; ^3^Epidemiology, Surgery, Universidad Surcolombiana, Neiva Huila, Colombia; ^4^Internal Medicine, Universidad Surcolombiana, Neiva Huila, Colombia

## Abstract

Venous thromboembolic disease is an important cause of mortality worldwide. A widely recognized risk factor is active neoplasia, mainly hematological tumors, in which associated thrombocytopenia can be a frequent complication. We present the case of a patient with submassive pulmonary thromboembolism associated with severe thrombocytopenia with signs of right heart failure and a requirement for systemic thrombolysis and anticoagulation, however with absolute contraindication for them. The case establishes a therapeutic challenge for the treating group, leading us to carry out an extensive search of the literature and propose a management algorithm in this complex situation.

## 1. Introduction

Venous thromboembolic disease (VTE) is part of the complex of venous thromboembolic disease that includes DVT (deep vein thrombosis) and pulmonary thromboembolism (PE). VTE is the third cause of death due to cardiovascular disease after ischemic heart disease and cerebral ischemia [1], affecting close to 100 cases per 100,000 people and with mortality up to 12% in the United States [2] and 14.8% in Colombia [3].

It is very important to identify the risk factors for VTE in all people; however it is described that the risk of thrombosis is up to seven times higher in cancer patients compared to the general population and recently it has been determined that the risk is higher in patients with cancer. Hematological neoplasia with respect to the rest of tumors is a frequent associated complication with the presence of thrombocytopenia [4].

Cancer is a major risk factor for ETEV but when we are faced with the association with thrombocytopenia that implies an additional risk of sacred, a therapeutic challenge is created for the treating group.

## 2. Clinical Case

We present the case of a female patient of 65 years of age, with a clinical picture of 4 days of evolution, consisting of syncopal episode, with subsequent development of dyspnea at rest, tachypnea, and malaise. One month prior to admission, she presented venous thrombosis of the lower right limb with involvement of the common femoral veins, superficial-deep femoral and popliteal veins, where thrombocytopenia had been additionally documented and for which she was under study. The patient had an indication for permanent anticoagulation with warfarin; she has anticoagulation so they start enoxaparin, which was not administered for 15 days. In addition to the aforementioned, she had a history of high blood pressure in treatment with enalapril, without any other relevant medical, family, or surgical history.

**Figure 1:**
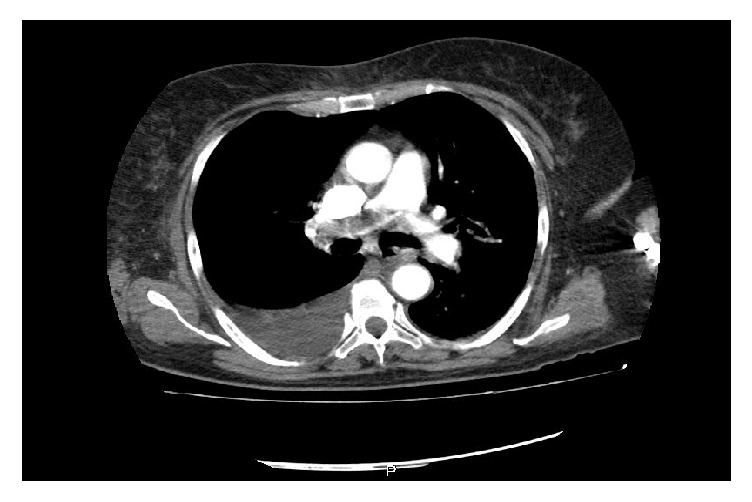
CT pulmonary angiography showing a large defect in the filling of the bifurcation and the right pulmonary artery.

The patient entered with marked respiratory distress and oxygen requirement at high flow without evidence of signs of shock or associated hypoperfusion. Within the initial studies (Table 1), leukocytosis called more bicytopenia due to anemia and severe thrombocytopenia, D dimer of 3030 mcg/l. This was equivalent to a high probability of pulmonary thromboembolism. Bilateral pulmonary arteriography showed large central intraluminal thrombus on right pulmonary trunk with obstruction of 70% extending to the right lobular artery, with evidence of angiography of the cloud microvascular on the same side without zones of oligohemia (Figure 1) and biomarkers of positive myocardial injury.

**Figure 2:**
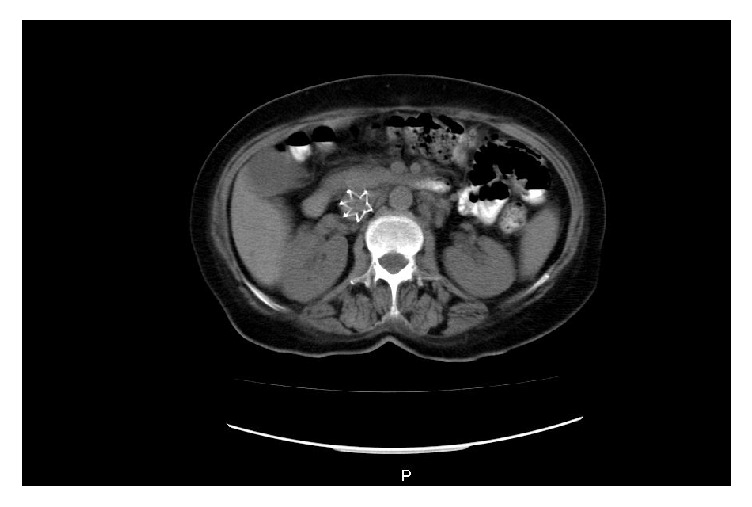
Vena cava filter.

**Figure 3:**
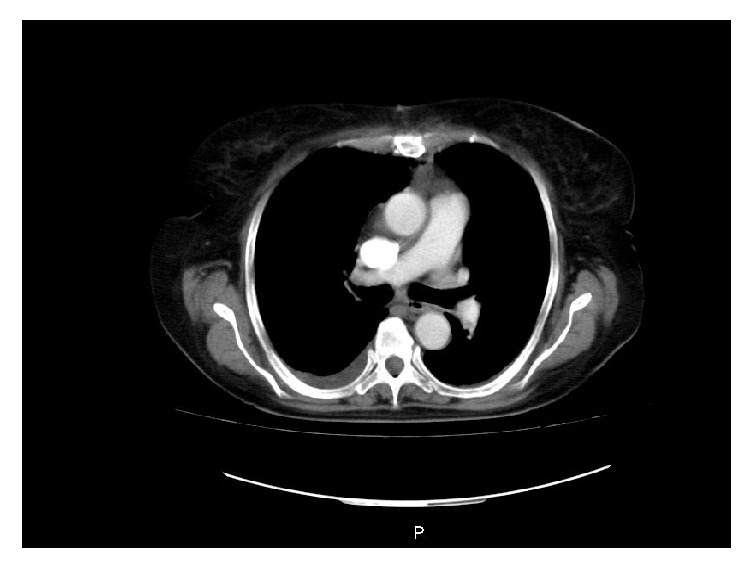
CT pulmonary angiography showing the bifurcation of the right pulmonary artery without clot.

The patient presented clear signs of heart failure, which is why it was considered the beginning of inotropia with levosimendan, immunoglobulin, and corticoides. At that time there was a clear indication for thrombolysis but an absolute contraindication for it, whereby placement of vena cava filter is ordered (Figure 2). Therefore, by means of an interdisciplinary meeting it is was decided to optimize the number of platelets and lead to endovascular embolectomy, a procedure that fails due to marked stiffness of the thrombus (right pulmonary trunk with obstruction of 90% that extends to the right lobular artery). One day later, obtaining a platelet count of 110,000, it was taken to open surgical embolectomy with good results (5 x 2 cms thrombus accommodation in the right of the pulmonary). A B cell lymphoma is documented as a cause of bicytopenia. Tomographic control is performed observing the absence of a clot at the level of the pulmonary artery trunk (Figure 3).

## 3. Discussion

The treatment of pulmonary thromboembolism depends on the severity, the risk of bleeding, and the specific considerations of the patient. The basis of treatment is anticoagulation, and according to the severity the additional management is determined [5].

The data regarding anticoagulation in thrombocytopenia are scarce and are based on expert recommendations. In the case of immune thrombocytopenic purpura that requires anticoagulation, it is recommended to optimize the number of platelets, starting anticoagulation at half the recommended dose if you have between 25,000 and 50,000 platelets and the full dose when the number exceeds 50,000 [6], with unfractionated heparin being the recommended medication given its short half-life and ease of reversion [7]. Recommendation was also indicated in patients with hematologic neoplasia and venous thromboembolism requiring anticoagulation [8]. In the case of the patient, the number of platelets was optimized using immunoglobulin, corticosteroid pulses, and platelet transfusion. We also report the use of argatroban in patients with thrombocytopenia associated with sepsis with adequate results [9], and cases of atrial fibrillation and thrombocytopenia associated with hematological neoplasms have been described where warfarin and rivaroxaban were safely administered without an increase in the risk of bleeding, before use of transfusions to maintain platelet level greater than 100,000 platelets [10]. The use of full dose was scheduled for 1 month followed by reduced dose for 3 months in patients with leukemia and has shown safety in 95% of patients [11].

Systemic thrombolysis is the treatment of choice in massive PE because it has shown a decrease in all causes of mortality, being quite high. In submassive PE, its use is still debated, having as a contraindication states of hemorrhagic diathesis [5, 12].

**Figure 4:**
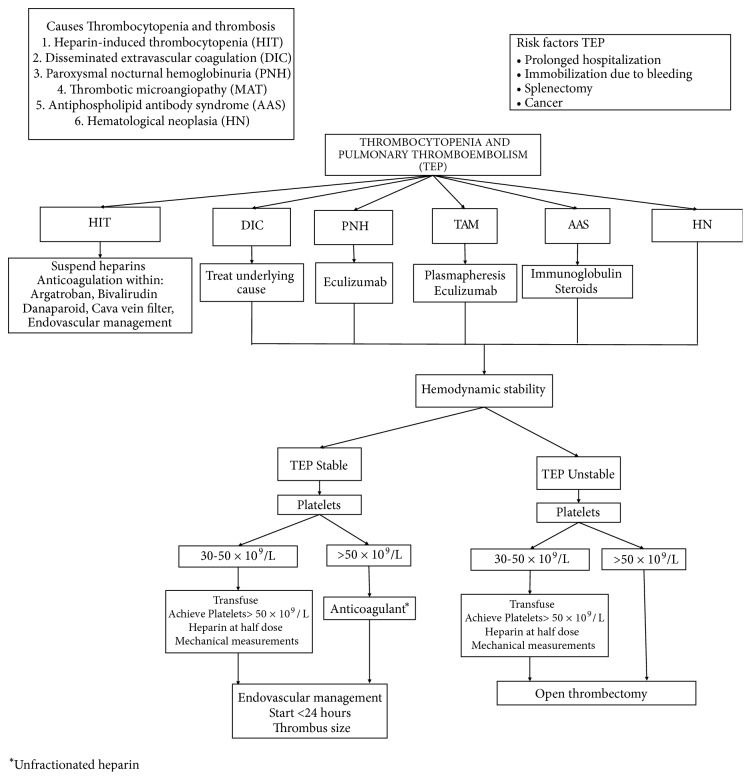
Algorithm for the management of pulmonary thromboembolism in a patient with associated thrombocytopenia.

There is no evidence on the use of thrombolysis in patients with thrombocytopenia [13]. Cases are reported in the literature where the use of endoluminal thrombolysis in patients with heparin-induced purpura thrombocytopenia did not increase the risk of bleeding [14, 15]. However, we did not find cases of use of embolectomy by aspiration or surgical embolectomy in this group of patients. With many questions and scarce literature on management protocols, we carried out a management algorithm which we propose as a new strategy that allows an easy classification of the safety and effectiveness of patients with PE and thrombocytopenia (Figure 4).

In the case of our patient, it is a question of submassive PE with imminent cardiovascular deterioration, absolute contraindication of systemic and local thrombolysis due to severe thrombocytopenia. Initial management generated an important debate about the initial use of anticoagulation. The patient was taken to endovascular management with aspiration thrombectomy, according to the current literature, the management most indicated by the condition of the patient.

## Figures and Tables

**Table 1:** Laboratory results.

Hemoleucogram	Leucocytes 19.8 10^3^x mm^3^ Neutrophils 33% Lymphocytes 59% Hemoglobin 10.3 g/dl Hematocrit 33.3 % VCM 82 fl Platelets 16 10^3^x mm^3^ (Manuals 18 10^3^x mm^3^). Manual Differential: 3% reactive lymphocytes, 56% lymphocytes, 41% neutrophils.

Blood smear	Anisocytosis - Microcytosis + - Normochromic. Peripheral Normal Morphology of Leukocytes and Platelets.

Kidney function	BUN 12.9 mg/dl, Creatinine 0.6 mg/dl

Liver function	Total Bilirubin 0.77 mg/dl, Direct 0.3 mg/dl, LDH 379 U/L, TP 12 seg, INR 1.1 seg, PTT 24.33 seg, Albumin 2,37 mg/dl, Fibrinogen 147 mg/dl, TGO 16,6 U/L, TGP 38 U/L.

Biomarkers of myocardial injury	Troponin 66 ng/l, D-Dimer 3030 mcg/l.

Arterial gasometry	PH 7.4 PCO2 26.7 PO2 144 HCO3 16.6 BE -6.9 PAFI 288 Lactate 0.93

Thromboelastogram	Compatible with procoagulant state
